# Interpersonal Change During Inpatient CBASP Treatment: Focus on Group Therapy

**DOI:** 10.3389/fpsyt.2021.620037

**Published:** 2021-02-26

**Authors:** Anne Guhn, David Schön, Yvonne Zische, Philipp Sterzer, Stephan Köhler

**Affiliations:** Department of Psychiatry and Psychotherapy, Charité – Universitätsmedizin Berlin, Corporate Member of Freie Universität Berlin, Humboldt-Universität zu Berlin, and Berlin Institute of Health, Campus Mitte, Berlin, Germany

**Keywords:** CBASP, group therapy, interpersonal problems, interpersonal style, change factors in group therapy, situational analysis, Kiesler's circle training

## Abstract

**Background:** The Cognitive Behavioral Analysis System of Psychotherapy (CBASP) has been tailored specifically to the demands of patients with persistent depressive disorder (PDD). According to the CBASP model, PDD patients are supposed to live perceptually disconnected from their social environment, which consequently maintains depression. While initially developed as an individual treatment modality, the adaptation for group therapy yields an important interpersonal space. However, little is known about the specific factors that contribute to patients' benefit from the CBASP group modality.

**Methods:** The analyzed sample comprised *N* = 87 PDD patients who completed a 12 week multimodal inpatient treatment including 2 weekly CBASP-specific individual and group sessions, respectively, as well as CBASP-unspecific medical contacts, pharmacotherapy and complementary therapies. Group sessions included trainings in situational analysis and interpersonal skills. Interpersonal change over therapy was examined based on the patients' self-perceived interpersonal problems (IIP) and the impact messages as perceived by their individual therapists (IMI). Pre and post-treatment data were compared using within-sample *t*-tests. Additionally, patients evaluated CBASP group therapy on a feedback form. They were invited to reflect on individual benefits and its helpful and unhelpful aspects. Qualitative content analysis with inductive category development was used to analyze feedback. Inter-rater reliability was computed to confirm categories before summarizing the frequencies of reported factors.

**Results:** Self-perceived interpersonal distress significantly decreased over therapy. Patients reported reduced interpersonal problems and therapists reported more friendly and dominant impact messages. Interestingly, patients who showed a significant depressive symptom reduction described higher change scores. Regarding qualitative data, patients reported five main benefits from group therapy: Gain in social competence, self-confidence, self-reflection, interpersonal dynamics, and optimism/universality. Patients responding to CBASP identified significantly more factors than non-responders.

**Conclusions:** Compared to studies with individual CBASP only, the present findings suggest that CBASP group therapy may contribute to the improvement of interpersonal behavior. Group therapy is discussed as a potential boosting effect for individual CBASP. However, as the present data were collected in a multimodal inpatient setting without competitor, randomized controlled trials are warranted that investigate the specific benefits of the group modality or the combined individual and group therapy over individual CBASP only.

## Introduction

Chronicity of depression is associated with high individual and economic disease burden (1). Compared to non-chronic forms of depression, pharmacotherapy and psychotherapy are less effective in patients with persistent depressive disorder [PDD, e.g., (2, 3)]. This may be due to specific features of PDD that impede treatment success, such as higher comorbidity rates and more avoidant, submissive and hostile interpersonal behavior (4). These risk factors are assumed to have their roots in childhood and require specific interventions. The Cognitive Behavioral Analysis System of Psychotherapy [CBASP; (5)] is an interpersonally oriented treatment approach specifically developed for the demands of patients with PDD (6). The CBASP model emphasizes childhood maltreatment as a cause of interpersonal dysfunctions that sustain chronicity of depression. Particularly emotional abuse and neglect by significant others during childhood elevate the risk of early-onset, severe, chronic and treatment-resistant depression (7–9). An unsafe or threatening home life is expected to redirect the normal cognitive-emotional development of a child toward survival rather than growth (5). Consequently, acquisition of behavioral, cognitive and emotional skills to build satisfying relationships later in life is impeded [e.g., (10)] and “primitive verbal thought and behavioral patterns serve to keep them perceptually disconnected from the environment” [(11) p. 834].

The interpersonal circumplex (IPC) model, which was developed in the middle of the last century (12), constitutes a useful tool for research and clinical practice to explain psychopathology within an interpersonal context. The model has two orthogonal axes which define a circular space that places normality and abnormality on a continuum (13). The vertical axis of *agency* represents the behavioral dimension of control that can range from dominance to submissiveness. The horizontal axis of *communion* represents the behavioral dimension of affiliation that can range from friendliness to hostility. Divided into eight segments that are arranged in equal increments (every 45°) around the circle, each octant represents a blend of these two axial dimensions and characterize a person's interpersonal profile. In contrast to non-clinical individuals, patients with depression show elevated levels of submissiveness and hostility (14–16) and PDD patients demonstrate even more hostile and less friendly interpersonal behavior than patients in acute depression (16, 17). Besides the characterization of the individual in the IPC space, the model incorporates dynamic transactional processes that continuously emerge between interaction partners. In terms of agency, dynamic transactions are reciprocal, whereas in terms of communion they are corresponding (18). Thus, submissive behavior of the chronically depressed patient invites dominant reactions by others, whereas hostile behavior evokes hostility in return. These principles of social interaction might account for the fact that PDD patients suffer from interpersonal problems and behavioral avoidance (19).

CBASP techniques intend to build (1) a feeling of interpersonal safety against the background of childhood adversity and (2) increase the patients' perceived social functionality. Therefore, CBASP therapists fulfill two central functions: They are to heal the interpersonal traumas patients have received in their significant other history by enacting a ‘Disciplined Personal Involvement Role’ (DPI); and they are to teach interpersonal skills in accordance with the dynamic transactional processes posited by the IPC model (20). Both these components of CBASP are supposed to (re)connect patients with their social environment. Realizing their interpersonal impact, patients are thought to become empowered to overcome submissiveness and hostility for the sake of acquiring satisfactory relationships with others. Thus, patients who respond to CBASP should change their interpersonal behavior from submissive to dominant and from hostile to friendly, corresponding to an increase in the dimensions of both agency (y-axis) and communion (x-axis).

In the outpatient and individual setting, CBASP has demonstrated efficacy in a growing number of randomized controlled trials (RCT) in PDD patients: CBASP proved to be as effective as medication (2, 21), particularly when CBASP was combined with medication (2); CBASP was more effective than psychotherapy as usual (22), Interpersonal therapy (23) and Supportive Psychotherapy (24), and yielded benefits particularly in patients with childhood maltreatment (25–27). CBASP has also been adopted and modified for the inpatient setting (28–30), which offers the possibility to combine individual and group treatment. CBASP group therapy is expected to boost the effects of individual therapy, since the relationships with other group members may promote interpersonal safety through an increased number of corrective interpersonal experiences. Group-CBASP may further provide a social network for exercising personal agency and communion, thereby fostering the patients' perceived functionality in the social domain. Previous studies have pointed to the feasibility (29) and effectiveness of CBASP group therapy regarding the reduction of depressive symptoms and the improvement of interpersonal functioning (17, 31–35), also as continuation therapy after acute treatment (36). However, little is known about the specific factors that contribute to patients' benefit from the group modality or a potential deterioration of symptoms that may be caused by the group setting itself. The present study aimed to elucidate interpersonal change within a naturalistic design by considering quantitative data on interpersonal functioning that derived from the entire multimodal CBASP inpatient setting and qualitative data that derived from the CBASP group modality in particular. Interpersonal change from pre- to post-treatment was evaluated from two perspectives. We expected that both, patients and their therapists would report a development from less agentic and less communal behavior at the beginning to increased agency and communion scores at the end of CBASP, particularly in responders. We also expected decreased levels of general distress in social interactions. Although limited to the naturalistic setting, which impedes strong conclusions with regard to the causal impact of CBASP group therapy, we further assumed the group to boost the effects of individual therapy. In our qualitative data, we therefore expected patients to identify CBASP-specific features when evaluating the specific benefits of group modality at the end of treatment.

## Materials and Methods

### Study Sample and Group Concepts

The present study was conducted at the general acute unit for affective disorders of the Department of Psychiatry and Psychotherapy of Charité Berlin (Campus Mitte), which offers a 12-week CBASP treatment for PDD patients. Ethical approval was obtained from the Ethics Committee of the Charité—Universitätsmedizin Berlin. Between April 2013 and August 2020, *N* = 105 patients were included in the CBASP program. Thereof, *n* = 18 were designated dropouts since they did not start therapy (*n* = 2) or discontinued therapy after fewer than 12 weeks. There was no difference between dropouts and completers regarding self-reported interpersonal problems at baseline (*t*_(17.4)_= 0.47, *p* = 0.641).

The present retrospective study reports data from *N* = 87 inpatients who completed a structured and manualized, multimodal inpatient CBASP concept (37), that is, dropouts are not considered. The majority of patients attended the first 6 weeks in an inpatient setting and the second half in a day-clinical setting on the same ward. Detailed descriptions of treatment components including pharmacotherapy, criteria of inclusion and exclusion as well as the effects of CBASP regarding the primary outcome, that is, depression change, were published elsewhere (30). In brief, the acute treatment consisted of 24 individual sessions and 24 group sessions. Besides 2 weekly individual sessions, patients attended 2 weekly manualized group therapies, one for the training of situational analyses [SA; (37)] and one for the training of interpersonal skills based on the IPC model, the so-called Kiesler's Circle Training [KCT; (38)]. Both groups originated from CBASP (5), but were adopted and modified for the inpatient setting and the group modality. They were half-open for three to eight patients at a time. SA group sessions lasted 100 min and were guided by a psychologist or psychiatrist trained in Cognitive Behavioral Therapy with further certification for CBASP therapy and training by the German national CBASP network (www.cbasp-network.de); KCT group sessions lasted 60 min and were guided by two masters-level psychologists in training for CBASP. Therapists got weekly supervision to guarantee adherence to the manuals.

The *SA training* constitutes the main skill acquisition exercise in CBASP. SAs were practiced during both CBASP individual and group sessions. During every group session, one patient mentions a conflictual interpersonal issue that he or she wants to analyze with the help of the other group members. The SA follows specific steps. The elicitation phase involves the description of the situation from an objective viewpoint followed by the interpretations that were involved during the situation. The protagonist of the SA is then encouraged to reflect on verbal and non-verbal behavior according to the interpersonal circle and the actual observable outcome this behavior entailed. This step is intended to make the patients appreciate their interpersonal impact and simultaneously serves to elucidate why they are left dissatisfied in social situations. The most important step comprises the specification of an interpersonal goal for the outlined situation, the so-called “Desired Outcome,” which needs to be realistic, attainable and under the protagonist's control. The end of the elicitation phase encompasses a comparison between the actual and the desired outcome. The subsequent remediation phase is intended to practice the achievement of the desired outcome. Dysfunctional interpretations are examined and transformed, complemented by an active interpretation. Subsequently, the patient's behavior is modified in theory and in practice. At the end of the group session, the therapist encourages every group member to derive a take home message from the protagonist's SA and to reflect on similar situations, in which the desired outcome of the present SA could be helpful for him- or herself. This is considered to facilitate learning transfer to similar conflictual interpersonal situations.

Within the KCT group, patients get familiar with the circumplex model and practice different techniques, which are taught in five modules: (1) Getting to know the circle, (2) non-verbal communication, (3) verbal communication, (4) conflict training, (5) empathy and corrective interpersonal experiences. Each session comprises a mixture of psychoeducational and experience-activating techniques to practice different interpersonal behaviors based on the octant IPC model. The KCT sessions do not follow a specific sequence due to the half-open group format; therapists rather select the topics according to the relevance for the group members at a specific time, so that KCT modules often complement SA training. For instance, patients learn to identify different adjectives that describe agency and communion (module 1), they learn to assign mimicry to the octant positions, (2) they try out how to actively express personal needs and demands (module 3), also in conflict scenarios (module 4), they reflect on individual experiences with other group members and learn to discriminate their reactions from former reactions with significant others (module 5). Handouts and worksheets support the consolidation of interpersonal learning.

### Quantitative Data

Change in interpersonal functioning over therapy was examined based on the patients' self-perceived interpersonal problems [IIP-64; (39)] and the impact messages as perceived by their individual therapists [IMI; (40)].

On the IIP-64, patients rate the extent to which a number of behaviors, thoughts and feelings in social interactions poses difficulties for them on 64 Likert-scaled items that range from 0 (not at all) to 4 (absolutely). The eight subscale scores (PA=domineering, BC=vindictive, DE=cold, FG=avoidant, HI=non-assertive, JK=exploitable, LM=overly-nurturant, NO=intrusive) cover the IPC space and reflect a particular combination of the interpersonal dimensions of agency and communion. The mean score of all 64 items further indicates the general level of interpersonal distress. Higher values represent more severe interpersonal problems. The psychometric properties of the German version of the IIP-64 are acceptable to good (41) and comparable to the English original version (42). Internal consistencies of the octant scales ranged from α = 0.71 to 0.88 and were slightly higher than in the present study (pre-treatment: α = 0.059 to 0.84, post-treatment: α = 0.68 to 0.88). However for self-reports in general, external validity may be reduced due to the limited ability to accurately characterize oneself with regard to interpersonal behavior. Therefore, we additionally assessed therapist-rated impact messages with the IMI. According to Altenstein-Yamanaka et al. (43), the agency scores of IIP-64 and IMI correlated moderately, whereas the communion scores did not, suggesting that others provide important additional information on interpersonal change.

On the IMI, observers rate the feelings, thoughts, and action tendencies evoked by a target person. For the purpose of the present study, individual CBASP therapists assessed their respective patients' interpersonal impact messages at week 2 and at the end of treatment. The IMI consists of 64 items rated on a 4-point Likert scale ranging from 1 (not at all) to 4 (very much). Paralleling the IIP-64, means of 8 items per octant are based on the underlying dimensions of agency and communion. In contrast to the IIP, higher values on the IMI indicate higher intensity of a specific octant, which does not necessarily entail more severe interpersonal problems. The psychometric criteria of the IMI demonstrated adequate psychometric and structural validation (44). Also the German IMI revealed acceptable internal consistencies of α = 0.68 to α = 0.97, both in a normative sample and in patients (40). Cronbach's alpha for the present IMI octant data was similarly satisfying, ranging from 0.85 to 0.91 at pre-treatment and 0.72 to 0.90 at post-treatment.

In accordance with earlier studies that have evaluated interpersonal functioning in depressed patients over therapy based on the IIP and/or IMI, we hypothesized decreased scores for all IIP-64 octant scores, so that also *general distress* would decrease over therapy (42, 43). For IMI, we considered that impact messages for dominant, friendly-dominant and friendly behavior (quadrant I) would increase, whereas hostile, hostile-submissive and submissive behavior (quadrant III) would decrease over therapy (17, 31, 45). We also considered that the reduction of general distress would be greater in patients who benefited from CBASP (43). Therefore, we considered self-reported depression scores measured at pre and post treatment using the Beck Depression Inventory [BDI, revised version; (46)]. We additionally differentiated between responders and non-responders, using a reduction of depressive symptoms by 50% as demarcation line.

All statistical analyses were performed with SPSS version 25. *N* = 85 patients answered IIP-64 at week 1 and *n* = 79 at week 12. Three individuals at week 1 and two individuals at week 12 missed the second page of the IIP-64 questionnaire. To keep the sample size constant, in these cases missing subscales for PA, JK, LM, NO were replaced by the group means, respectively. The impact messages (IMI) of *n* = 83 patients were assessed by the individual therapists at both pre and post treatment.

Changes over therapy were analyzed using paired *t*-tests. To protect against type I error due to multiple comparisons, we set the cut-off value for significance at *p* ≤ 0.003 and calculated effect sizes according to Cohen (1992), that is, *d* ≤ 0.2 represents small, *d* = 0.5 medium and *d* ≥ 0.8 large effects. In accordance with the CBASP model we further associated treatment response with interpersonal change (5). Therefore we calculated Spearman correlation coefficients between BDI change (BDI_change_ = BD_pre_−BDI_post_/BDI_pre_) and change in IIP general distress (IIP-64 = distress_pre_-distress_post_/IIP-64_pre_), corrected for the respective baseline values. Missing data for BDI (at pre: *n* = 13, at post: *n* = 12; that is, 14.4% missing values) were replaced by a multiple imputation with five iterations since data were confirmed to be completely random (little MCAR test: ${\text{Chi}}_{\left(2\right)}^{2}=$ 4.6, *p* = 0.103; *n* = 3 patients neither had pre nor post BDI scores). Imputed and observed results showed comparable values, so that pooled imputed values are reported. This resulted in a subsample of *n* = 76 complete data sets for the above mentioned correlation analysis between interpersonal functioning and severity of depression.

### Qualitative Data: Evaluation Form

At the end of treatment, patients evaluated their experiences with CBASP group therapy on a feedback form, which consisted of a shortened and modified version from Brakemeier, Strunk, Normann and Schramm used in the study by Sabaß et al. (29). Amongst others the feedback form comprised quantitative measures regarding patients' motivation and engagement in group therapy as well as an overall grade for the group using the German educational grading system (1 = “very good” to 6 = “insufficient”). Additionally, patients were invited to reflect on (1) what they have learned throughout group-CBASP in retrospect, and (2) what they experienced as helpful and (3) not helpful, respectively, through three qualitative measures, which constitute the main focus of the present study. These items were analyzed according to the procedures of the qualitative content analysis with inductive category development (47). In contrast to deductive category development where categories originate from existing theoretic models or data, inductive categories derive out of the text. First, the material is step-by-step divided into content analytic units. Out of these units, subsequently categories are generated by formulating a criterion of definition. In the present study, one author (YZ) first deduced the formulation of categories, while in a second step, these categories were revised within a feedback loop with a second author (AG). Both raters then worked through the material independently and analyzed the content units a second time by following the final set of criteria for categorization (see Supplementary Table). The inter-coder reliability for question one (259 units) was Cohen's κ = 0.63, for question two (142 units) κ = 0.78 and for question three (68 units) κ = 0.79, that is, the overall inter-coder reliability was κ = 0.73, demonstrating sufficient intersubjective comprehensibility (47). Finally, both raters discussed their mismatches to agree on the respective category for each unit that matched the criterion best, so that quantitative aspects concerning the frequencies of coded categories could be analyzed. Paralleling the quantitative data analyses, responders and non-responders according to BDI change were compared with regard to the frequency of individual profits during group therapy (question one).

## Results

### Sample Characteristics

A total of *N* = 87 patients (44 men, 43 women) with a mean age of 44.2 years (±10.8) participated in the study. Twenty-six patients were employed (30%), the majority was either unemployed (35%), in early retirement (24%), retired (5%) or in educational training (6%). Fifty-four patients (62%) lived without partner. Sixty patients (69%) reported an early onset of depression (<21 years) with a mean age of 16 years (±9.2), 11 patients (13%) did not remember the beginning of depressive symptoms. All patients fulfilled the criteria for PDD. Comorbid diagnoses are limited to information obtained from discharge letters, since we did not carry out structured clinical interviews. This may explain why only 25 patients (29%) were treated for a comorbid diagnosis, such as alcohol dependence with more than 6 months abstinence (*n* = 7), panic disorder with/without agoraphobia (*n* = 6), bulimia nervosa (*n* = 3), attention-deficit-(hyperactivity)-disorder (*n* = 3), psychosomatic disorders (*n* = 2), social phobia (*n* = 1), obsessive compulsive disorder (*n* = 1), enuresis nocturna (*n* = 1). One patient was diagnosed with Alzheimer's disease during CBASP treatment. According to the self-assessment of personality disorders according to DSM-IV [ADP-IV; (48)], the majority of patients (69%) fulfilled the criteria for at least one comorbid personality disorder, particularly in cluster C (avoidant, dependent, obsessive-compulsive). The frequency of childhood adversity was high (mean CTQ total score = 54.6±16.5), particularly concerning emotional neglect (mean subscale score = 16.6±5.7) and emotional abuse (14.1±5.4), whereas physical neglect (9.7±3.8) and abuse (8.4±4.5) were rather low to minimal; sexual abuse was mentioned very rarely (5.8±2). The majority of patients (86 %) had at least moderate to severe traumatization in one out of five subscales (49).

Regarding treatment outcome, patients reported severe depressive symptoms at pre-treatment (BDI_pre_= 32.8±10.3), which decreased significantly to post-treatment (*t*_(83)_= 7.0, *p* <0.001, *d* = 0.8), although BDI scores remained on a moderate level (BDI_post_= 22.9±13.3). Twenty-seven patients (32.1%) showed a significant response as demonstrated by a reduction of 50% from pre-treatment BDI score.

### Quantitative Data: IIP-64 and IMI

**Figure 1:**
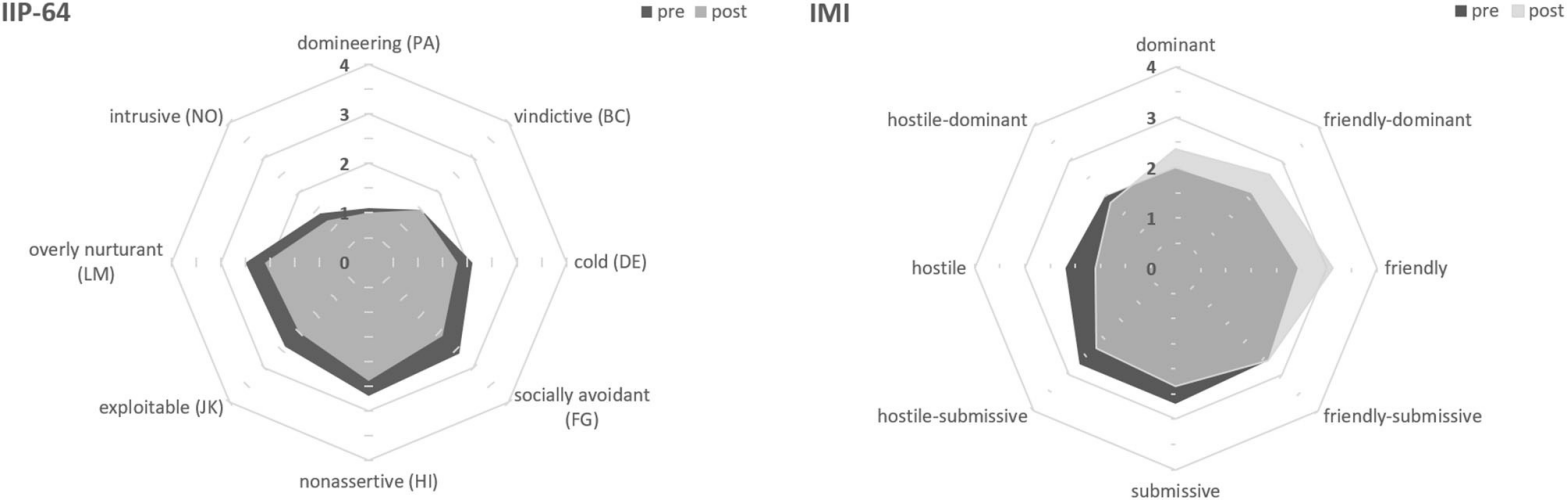
Circumplex models of the IIP-64 and IMI octants. Pre-treatment values (dark gray) represent interpersonal problems as perceived by patients (IIP-64, left) and impact messages as perceived by their therapists (IMI, right). Post-treatment values (light gray) indicate change over therapy.

As predicted, patients showed a significant reduction in general distress over therapy (*t*_(77)_= 4.9, *p* <0.001, *d* = −0.6; see Table 1). Regarding the IIP-64 octant scales, patients reported reduced interpersonal problems with most of the octant scales after therapy, except for domineering (PA) and vindictive behavior (BC) where patients posed almost no difficulties with at both time points. Therapists also perceived significant changes from pre to post treatment with values increasing particularly in IMI quadrant I (dominant, friendly-dominant, friendly) and decreasing particularly in quadrant III (hostile, hostile-submissive, submissive) of the IPC, as predicted. Table 1 provides descriptive and change statistics with effect sizes for all octant subscale scores; Figure 1 depicts the according graphical illustration on the IPC model.

**Table 1:** IIP-64 and IMI subscale scores (M, SD).

**IIP-64 subscales**	**pre (*n* = 85)**	**post (*n* = 79)**	**Statistics**	**Cohen's *d***
PA—domineering	1.1 (0.6)	1.0 (0.6)	*t*_(77)_=1.8, *p* = 0.079	−0.2
BC—vindictive	1.5 (0.6)	1.5 (0.7)	*t*_(77)_=1.4, *p* = 0.156	−0.2
DE—cold	2.1 (0.8)	1.8 (0.8)	*t*_(77)_=3.9, *p* <0.001^*^	−0.4
FG—avoidant	2.6 (0.8)	2.1 (0.8)	*t*_(77)_=4.8, *p* <0.001^*^	−0.5
HI—non-assertive	2.7 (0.8)	2.4 (0.7)	*t*_(77)_=3.4, *p* = 0.001^*^	−0.4
JK—exploitable	2.4 (0.6)	2.0 (0.7)	*t*_(77)_=4.8, *p* <0.001^*^	−0.6
LM—overly nurturant	2.5 (0.6)	2.1 (0.7)	*t*_(77)_=4.5, *p* <0.001^*^	−0.6
NO—intrusive	1.4 (0.7)	1.2 (0.6)	*t*_(77)_=3.5, *p* = 0.001^*^	−0.3
General distress	2.0 (0.4)	1.8 (0.5)	*t*_(77)_=4.9, *p* <0.001^*^	−0.6
**IMI subscales**	**pre (n** **=** **83)**	**post (n** **=** **83)**	**Statistics**	**Cohen's** ***d***
Dominant	2.0 (0.6)	2.4 (0.5)	*t*_(80)_=-6.1, *p* <0.001^*^	0.6
Friendly-dominant	2.1 (0.6)	2.7 (0.5)	*t*_(80)_=-8.6, *p* <0.001^*^	0.9
Friendly	2.4 (0.5)	3.2 (0.5)	*t*_(80)_=-9.8, *p* <0.001^*^	1.2
Friendly-submissive	2.5 (0.6)	2.6 (0.4)	*t*_(80)_=-0.8, *p* = 0.446	0.1
Submissive	2.7 (0.7)	2.3 (0.6)	*t*_(80)_=5.5, *p* = 0.001^*^	−0.6
Hostile-submissive	2.7 (0.6)	2.2 (0.6)	*t*_(80)_=6.6, *p* <0.001^*^	−0.7
Hostile	2.2 (0.6)	1.6 (0.5)	*t*_(80)_=7.7, *p* <0.001^*^	−0.9
Hostile-dominant	2.0 (0.7)	1.8 (0.5)	*t*_(80)_=2.7, *p* = 0.009	−0.3

* *Significant result (p-value ≤ 0.003, Bonferroni-corrected)*.

Paralleling these results, change in general distress and change in depression severity showed a moderate association over all patients (*r*_*s*__(76)_ = 0.57, *p* <0.001), that is, the higher the symptom improvement was from pre to post-treatment the higher was the reduction of interpersonal distress or vice versa.

### Group Evaluation

Out of the investigated sample, *n* = 69 patients (79%) answered the feedback form at the end of treatment. They reported satisfying motivation (M = 2.0±0.9) and engagement (M = 2.2±0.9) for group therapy and evaluated the group with an overall grade of 1.7 (±0.7) indicating very good acceptance.

#### “What Have You Learned Throughout Group Therapy?”

**Figure 2:**
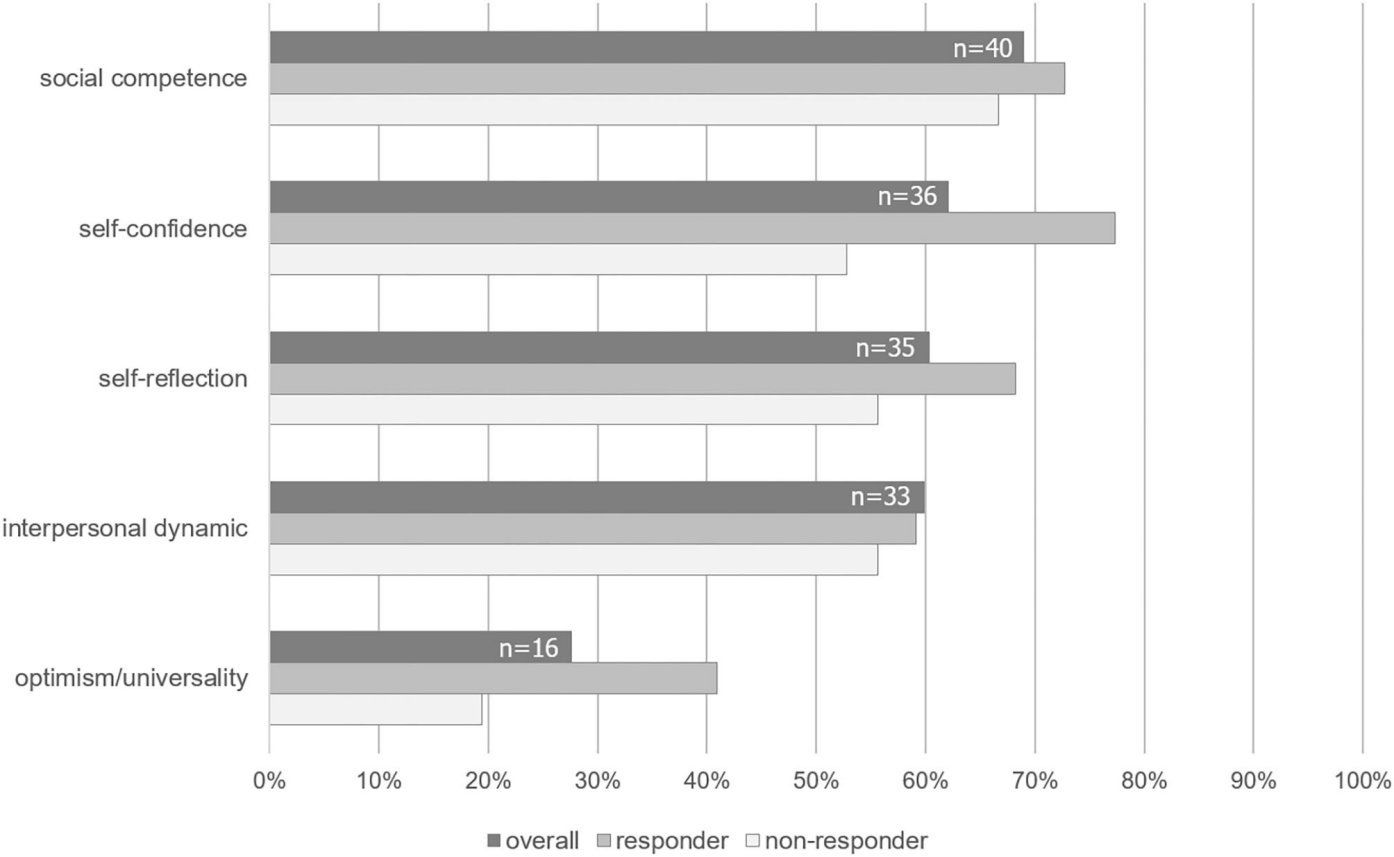
Frequencies of categories mentioned as benefit from group therapy derived from qualitative data. Dark gray bars depict the overall sample of *n* = 58 PDD patients, who answered the feedback form (question 1). Lighter bars represent the frequencies of categories identified by responders and non-responders to CBASP according to BDI. Note that frequencies differ due to the open format of the questionnaire.

From *n* = 58 patients who answered question 1 of the feedback form, 259 content units were derived for qualitative content analysis. These units comprised the following specific aspects, which were defined to facilitate allocation to content categories: Patients differentiated between acquisition of skills (active) and gain in knowledge (passive), both of which concerned either themselves as individuals (intrapersonal) or their interactions with others (interpersonal). Following these aspects, five inductive content categories were derived (Supplementary Table 1): (a) *social competence*, (b) *self-confidence*, (c) *self-reflection*, (d) *interpersonal dynamics*, and (e) *optimism/universality*. Accordingly, we specified content units that matched the category *self-reflection* to represent a progress in insights to personal needs as passive and intrapersonal, while a progress in *social competence* represented a combination of active and interpersonal aspects. Concerning the number of categories (Figure 2), the most frequent answers delineated gain in *social competence* (reported from 69% of patients), followed by gain in *self-confidence* (62.1%) and *self-reflection* (60.3%). As expected, patients who responded to CBASP (ΔBDI≥50%, *n* = 22) reported significantly more individual benefit (M = 3.2±0.9 out of five categories) than those with response rates of <50% (*n* = 36, M = 2.5±1.1) from baseline (*U* = 253.5, *Z* = −2.4, *p* = 0.02). Importantly, data availability was not biased by response to treatment, i.e. responders and non-responders did not differ in providing feedback at all ($\chi_{\left(1\right)}^{2}$ = 1.6, *p* = 0.207).

#### “What Was Particularly Helpful or Unhelpful During Group Therapies?”

Fourty-nine patients made comments with regard to particularly helpful aspects of CBASP group therapy, *n* = 40 patients commented on negative aspects (Supplementary Table 2).

Six categories regarding helpful factors were derived from content analysis, that is, *specific CBASP techniques* (role play, SA, IPC model) mentioned by *n* = 30 patients (61.2%)*, working atmosphere and therapeutic competence* (*n* = 25, 51%), *group cohesion* (*n* = 22, 44.9%)*, individual progress* in the behavioral domain (*n* = 13, 26.5%), *feedback* (*n* = 12, 24.5%), and *handouts and work sheets* (*n* = 2, 4.1%).

Regarding the factors that impeded progress during group therapy, patients mentioned *conceptual issues* mostly regarding time limits (*n* = 36, 90%), *outside disturbances* (*n* = 15, 37.5%)*, deficits in group cohesion* (*n* = 6, 15%)*, doubts upon the CBASP concept* (*n* = 3, 7.5%) and *group size* (*n* = 2, 5.6%). Concerning the main category *conceptual issues*, some patients criticized a lack of introduction to the group that felt like throwing them into cold water [*direct quotation*]. These patients would have wished for individual sessions prior to entering group therapy. Another issue comprised the long duration of the SA training group; three patients reported having felt pressured to provide enough material for the SA group.

## Discussion

The present study investigated interpersonal change during a multimodal inpatient CBASP treatment with a special focus on CBASP group therapy. Since the goals of CBASP group therapy parallel those described for individual therapy, the group was expected to boost the treatment effects regarding (1) felt interpersonal safety and (2) perceived functionality. In a nutshell, the group should counter-condition the interpersonal fear of emotional neglect and abuse rooted in the PDD patients' learning history. Both treatment goals aim at (re)connecting patients with their social environment, since overcoming social isolation is considered indispensable for the successful treatment of chronic depression (5).

As derived from our quantitative and qualitative data in a naturalistic setting, CBASP group therapy may contribute to the improvement of interpersonal functioning in PDD patients. Patients reported reduced general distress after therapy, which may indicate reduced feelings of hopelessness as proposed by the CBASP model. They described themselves as having less problems in the domains of submissiveness and hostility, which paralleled the patients' impact messages on their therapists. As predicted, patients changed from submissive and hostile interpersonal style to more friendly and dominant behavior.

Since the effects of group therapy on these quantitative interpersonal changes could not be separated from those of individual therapy in our treatment setting, we additionally asked patients to reflect on competencies learned specifically during CBASP group therapy. From the patients' perspective, five factors contributed to the benefit of group therapy: Gain in social competence, self-confidence, self-reflection, interpersonal dynamics, and optimism/universality. These factors were derived from inductive qualitative content analysis, but can be related to common factors in group therapy identified in quantitative research (50), that is, social learning, secure emotional learning, awareness of relational impact, and installation of hope. The majority of our PDD patients reported gain in social competence throughout CBASP group sessions. We suggest that the highly structured skill training within SA and KCT group, made social competence the most frequently mentioned benefit of CBASP group therapy. This interpretation of social competence as specific change factor to CBASP is limited by our naturalistic design, but in accordance with Klein et al. (51), who found patients receiving CBASP to exhibit significantly greater gains in social problem solving and positive problem orientation than patients receiving brief supportive therapy. The second and third frequent categories, self-confidence and self-reflection, refer to intrapersonal processes of the individual as a group member and may indicate that CBASP patients started to reflect on themselves as part of a social network, therewith overcoming the cycle of isolation. An insight into interpersonal dynamics the fourth change factor, can be regarded as necessary precondition to approach social interactions. Again, interpersonal dynamics might be a common factor, but it may also be specific to CBASP, since CBASP therapists provide explicit feedback to their patients (DPI) and encourage fellow patients to provide feedback as well. The fifth change factor identified in the present study was optimism/universality. It incorporates a sense of belonging and acceptance by others. Although the installation of hope is regarded a common factor in group therapy, it is remarkable that PDD patients draw strengths from a group, although they are supposed to live disconnected from others, which may explain the high rate of patients living without partner in the present sample. This may indicate progress in felt interpersonal safety, one of the two treatment goals in CBASP.

Considering the CBASP model, identification of change factors should contribute to positive treatment outcome. Accordingly, patients who responded to CBASP identified significantly more beneficial factors than non-responders, particularly with regard to self-confidence and self-reflection, and CBASP benefit was related to less interpersonal distress as provided by the CBASP model (5). Obviously, this finding may be biased given the fact that responders might have expressed their gratitude to therapists by filling in the evaluation form. However, responders were not more likely to provide feedback than non-responders.

The reported factors that boosted or impeded the effect of group therapy both surprisingly comprised group cohesion amongst others. On one hand, a sense of belonging seems to match the need for social contacts of PDD patients, on the other hand a lack of group cohesion may increase distrust and impede progress in CBASP goals. Leading group therapies thus places high demands on CBASP therapists. Accordingly, patients evaluated the therapists' expertise as second most helpful aspect. This category included clear instructions, for instance while doing SAs, and active inclusion of all group members. Most units of this category related to the DPI principles, that is, patients appreciated to be promoted and challenged at the same time, and described a feeling of safety within the group due to the actively communicated empathy including constructive feedback and the establishment of group rules by the therapists. CBASP therapists' expertise probably promoted another common factor of psychotherapy, that is therapeutic alliance (52), which in turn may have contributed to interpersonal functioning [see (53, 54).

Major limitations of the present study need to be discussed. Interpersonal change during CBASP inpatient treatment considering particularly the feasibility and efficacy of CBASP group therapy comprised the main focus of our study; however, group therapy was only one out of several treatment components. Although we consider CBASP group therapy very important for PDD patients, the present data must be regarded as reflecting a combined influence of the individual and group modality on interpersonal functioning. For comparison, Constantino et al. (17) found lower effect sizes for IMI changes particularly concerning friendly, dominant, hostile, and friendly-submissive behavior in outpatients receiving 16 individual CBASP sessions, suggesting superior effectiveness of the inpatient setting including group therapy on interpersonal functioning. However, it should be noted that, besides CBASP group therapy, inpatient treatment entails other ingredients that might have positively influenced outcome. Future studies are warranted that investigate the benefits of combined individual and group CBASP therapy over individual CBASP in a randomized controlled design. Furthermore, CBASP group therapy was evaluated in a naturalistic design without an active competitor. Thus, although it is tempting to trace the qualitative and quantitative results on interpersonal functioning back to CBASP (51), it is an open question whether a treatment irrespective of the CBASP model would have obtained similar results. In this regard, unspecific effects of the inpatient setting such as treatment duration [e.g. (55, 56)] might also account for the reduction of general distress. Results from previous studies conducting other interventions than CBASP were heterogeneous with regard to effects on interpersonal functioning: Cognitive interventions yielded effect sizes that were comparable to our results (57) or even larger (58) while effects of interpersonal psychotherapy were smaller (59). Notably, the present inpatient sample suffered from a higher degree of interpersonal problems than the outpatients included in these previous studies, which limits comparability. Future studies should compare interventions directly to differentiate intervention-specific from more general treatment effects on interpersonal functioning. A further limitation refers to the lack of a structured diagnostic interview to characterize the sample with regard to comorbid disorders. Importantly, but limited to self-report, there was a high comorbidity with personality disorders. It is tempting to speculate that the proposed group interventions for situational analysis and Kiesler's Circle Training may be similarly advantageous for patients with personality disorders irrespective of a PDD diagnosis. Future studies should investigate the benefit of both group therapies also for other diagnosis with interpersonal problems. Independent raters who evaluate interpersonal behavior from videotaped therapy sessions [e.g., (60)] or raters who are familiar to the patient, but not related to the therapeutic process, such as partners or friends [e.g., (43, 45)] may further increase the validity of the observed interpersonal changes.

To summarize the present results, we conclude with recommendations for CBASP group therapy while considering helpful and unhelpful factors identified by CBASP inpatients:

1. Patients should be prepared for entering into CBASP group therapy in individual sessions. CBASP therapists should encourage patients to reflect on concerns or fears of the group with regard to the individual significant other history (e.g., “When I disclose my feelings, other group members will laugh at me.”). These concerns are suitable for defining a specific treatment goal for the group (e.g., “I will learn to express my feelings.”), which will increase the number of corrective interpersonal experiences.

2. CBASP therapists should support patients to actively practice CBASP techniques (SA, interpersonal circle), since patients benefitted from increased social competence the most. Therefore, role plays, work sheets and transcripts during group sessions seem important.

3. Therapists should be aware of group cohesion by considering specific stages of group development [cf. (61)]. They should actively integrate rather than force more submissive group members to contribute to the group and should be aware of interpersonal situations arising within the group to apply SA and IPC techniques, therewith demonstrating their effectiveness in coping with conflictual interpersonal situations within the group.

## Data Availability Statement

The raw data supporting the conclusions of this article will be made available by the authors, without undue reservation.

## Ethics Statement

The studies involving human participants were reviewed and approved by Charité's Ethics Committee, Charitéplatz 1, 10117 Berlin. The patients provided their written informed consent to participate in this study.

## Author Contributions

AG: conceptualization and implementation of the study design, data collection, data analyses, and writing the original draft. DS: quantitative data analysis and editing the draft. YZ: qualitative data analysis and editing the draft PS: conceptualization and implementation of the study design, supervision of data analyses, editing the draft, revision. SK: conceptualization and implementation of the study design, funding acquisition, and editing the draft. All authors contributed to the article and approved the submitted version.

## Conflict of Interest

AG, PS, and SK received honoraria for workshops and presentations relating to CBASP. The remaining authors declare that the research was conducted in the absence of any commercial or financial relationships that could be construed as a potential conflict of interest.
