# Supplementary material for: Interpersonal Change During Inpatient CBASP Treatment: Focus on Group Therapy
Source: Front Psychiatry. 2021 Feb 26;12:620037. doi: 10.3389/fpsyt.2021.620037 (PMC7952983; doi:10.3389/fpsyt.2021.620037)
Supplement: Supplementary file 1 [file Data_Sheet_1.pdf]

Supplement Material

Guhn, A., Schön, D., Zische, Y., Sterzer, P. & Köhler, S. (2021). Interpersonal change during inpatient CBASP treatment: Focus on group therapy. *Front. Psychiatry 12*:620037. doi: 10.3389/fpsyt.2021.620037

Table 1. Main categories of learned aspects through group therapy derived from qualitative data. All examples are direct quotes from patients, translated from German to English by the authors.

	Category	Definition	Focus		Examples
1	**Interpersonal dynamics**	Awareness that behavior has consequences on others	Interpersonal	passive	*I realized that social interactions are interrelated.*<br>*I can actively influence how other people treat me by changing my behavior.*<br>*I became more aware of interactions between people.*
2	**Social competence**	Aquisition of social skills, e.g. disclosing feelings	Interpersonal	active	*I learned how to express what I need.*<br>*I learned how to practically apply the theoretical knowledge I learned in Kiesler's Circle Training in contact with other people (role plays!).*<br>*The group offered a good opportunity to practice how to act more self-confident.*
3	**Self-confidence**	Increase in confidence through self-acceptance or the acquisition of skills, e.g. SA	Intra- and interpersonal	active and passive	*The SA structure helped me to identify my personal goals.* (active)<br>*I found the courage to act more flexible and to leave familiar paths.* (active)<br>*I learned to endure or change unbearable situations.* (active)<br>*I learned to listen to my feelings.* (passive)<br>*I don't have to justify myself any longer*. (passive)<br>*I learned to trust in myself.* (passive)
4	**Self-reflection**	Insights to individual needs, also against the background of the significant other history	intrapersonal	passive	*I realized that my thinking is often biased by my stamps* [significant other history].<br>*I learned to analyze my thoughts and feelings.*<br>*I realized that my behavior is rooted in my childhood, and that it is no longer useful today.*
5	**Optimism & universality**	Feelings of hope and a sense of belonging (group cohesion)	interpersonal	passive	*There is at least one solution for every problem.* (optimism)<br>*Many symptoms of depression can be alleviated.* (optimism)<br>*I have experienced support and understanding.* (optimism)<br>*Others have similar problems and feelings.* (universality)<br>*Often, I was able to identify myself with the others and no longer felt so alone with my problems, "We-feeling".* (universality)<br>*I am part of a group.* (universality)

Supplement Material

Guhn, A., Schön, D., Zische, Y., Sterzer, P. & Köhler, S. (2021). Interpersonal change during inpatient CBASP treatment: Focus on group therapy. *Front. Psychiatry 12*:620037. doi: 10.3389/fpsyt.2021.620037

Table 2. Helpful (left) and non-helpful (right) aspects of group therapy derived from qualitative data.

	Category	Definition and examples *(direct quotes)*
1	**Techniques**	Role play, SA, etc.<br>(*Role plays helped me to change my behavior*)
2	**Working atmosphere, therapists' competence**	Standard of work between group members and Instructions by therapists<br>(*It was helpful for me that therapists were absolutely convinced of CBASP*)
3	**Group cohesion**	Feelings of interpersonal safety, support, and belonging<br>(*Mutual assistance and support in the group*)
4	**individual progress**	Realization of behavioral progress throughout therapy<br>(*To see my behavior enacted by someone else*)
5	**Feedback**	Receiving feedback from therapists and fellow patients<br>(*I got a lot of feedback about the impact of my behavior on others and how to change this to achieve my desired outcome*)
6	**Handouts and worksheets**	All materials that illustrates group contents, such as work sheets helping to conduct SAs, Kiesler's Circle, etc.<br>(*Flip chart notes were vivid and helpful*)

	Category	Definition and examples *(direct quotes)*
1	**structural issues**	Problems of comprehension due to time limits, a low frequency of sessions, insufficient instructions, missing room for open discussions between group members<br>*(There is too much emphasis on SAs, therapy could be more flexible)*
2	**Outside disturbances**	Disturbances regarding the general acute unit<br>*(extremely noisy and chaotic ward, a separate CBASP space needs to be created urgently)*
3	**Deficits in group cohesion**	missing sense of belonging, submissive and hostile fellow patients, insufficient motivation of group members, not respecting rules<br>*(There is a missing input and group pulse if other members are little or not engaged)*
4	**Doubt upon CBASP**	Doubts upon CBASP techniques for long-lasting or sufficient reduction of depressive symptoms<br>*(I think CBASP is effective for interpersonal communication; however, I don't believe it will positively influence my depression)*
5	**Group size**	Not enough fellow patients to create a working atmosphere<br>(*Sometimes, there could have been more participants in the group*)

Note: Direct quotations are displayed in italic font.
